# Endoscopic decompression for intraforaminal and extraforaminal nerve root compression

**DOI:** 10.1186/1749-799X-6-16

**Published:** 2011-03-26

**Authors:** Toshio Doi, Katsumi Harimaya, Yoshihiro Matsumoto, Osamu Tono, Kiyoshi Tarukado, Yukihide Iwamoto

**Affiliations:** 1 Department of Orthopaedic Surgery, Graduate School of Medical Sciences, Kyushu University, Fukuoka, Japan

## Abstract

**Objective:**

The purpose of this study was to evaluate the outcome of endoscopic decompression surgery for intraforaminal and extraforaminal nerve root compression in the lumbar spine.

**Methods:**

The records from seventeen consecutive patients treated with endoscopic posterior decompression without fusion for intaforaminal and extraforaminal nerve root compression in the lumbar spine (7 males and 10 females, mean age: 67.9 ± 10.7 years) were retrospectively reviewed. The surgical procedures consisted of lateral or translaminal decompression with or without discectomy. The following items were investigated: 1) the preoperative clinical findings; 2) the radiologic findings including MRI and computed tomography-discography; and 3) the surgical outcome as evaluated using the Japanese Orthopaedic Association scale for lower back pain (JOA score).

**Results:**

All patients had neurological findings compatible with a radiculopathy, such as muscle weakness and sensory disturbance. MRI demonstrated the obliteration of the normal increased signal intensity fat in the intervertebral foramen. Ten patients out of 14 who underwent computed tomography-discography exhibited disc protrusion or herniation. Selective nerve root block was effective in all patients. During surgery, 12 patients were found to have a protruded disc or herniation that compressed the nerve root. Sixteen patients reported pain relief immediately after surgery.

**Conclusions:**

Intraforaminal and extraforaminal nerve root compression is a rare but distinct pathological condition causing severe radiculopathy. Endoscopic decompression surgery is considered to be an appropriate and less invasive surgical option.

## Background

Intraforaminal and extraforaminal nerve root compression at lumbar lesions is much rarer than intraspinal canal lesions, making the diagnosis difficult [1]. The difficulties in making a correct diagnosis could unfortunately result in a failed lumbar spine surgery.

Surgical intervention is considered for patients with severe radiculopathy that does not respond to conservative treatment. As interverebral foraminal nerve entrapment mostly affects the elderly, it is better to choose a minimally-invasive surgical procedure. Consequently, we have treated patients with intraforaminal and extraforaminal nerve root compression by posterior decompression surgery without fusion. This procedure can obtain clear visualization of the deep surgical field with minimal damage to the posterior lumbar structure [2-5].

The purpose of this study was to elucidate the radiological findings, including those obtained by computed tomography-discography, and the surgical outcomes and limitations of posterior decompression without fusion for this relatively uncommon disease.

## Methods

Seventeen patients (7 males and 10 females, mean age at the time of surgery: 67.9 ± 10.7 years, range: 40 - 88 years) with intervertebral foraminal entrapment were treated by endoscopic posterior decompression at Kyushu University Hospital from 2008 to 2010. The mean follow-up period was 10.8 moths (range: 4 - 20 months). All patients had leg pain that did not respond to conservative treatment, such as NSAIDs, epidural steroid injection or the use of a brace. Three patients had a history of previous lumbar surgery, including posterior decompression of the spinal canal. They had experienced only slight improvement after the previous surgeries.

A diagnosis of intraforaminal or lateral entrapment was established in each patient based on the results of both neurological and radiological examinations. MR imaging were performed in all patients. We performed CT discograms for all but 3 patients to describe the positional relationship of the protruded disc to the posterior elements, such as the facet, transverse process and pars interarticularis, and such information was very useful for selecting the optimal surgical approach. Selective nerve root blocks, using 0.8 ml of 1% lidocaine, were also performed.

### Surgical procedures

**Figure 1:**
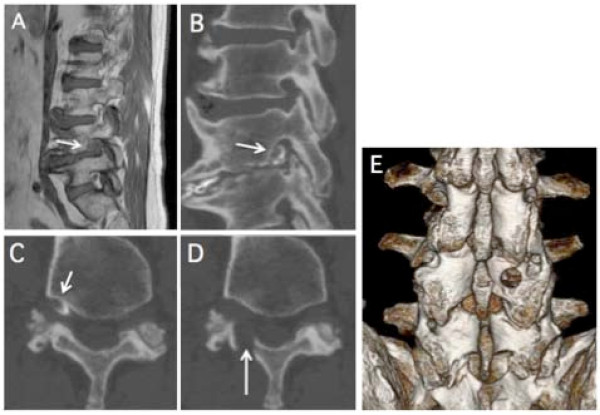
**Case 6. Images from a 56-year-old male who presented with severe right leg pain**. A: Para-sagittal MRI imaging (T1) showed the obliteration of the normal increased signal intensity fat (arrow). B,C: Para-sagittal and axial reconstruction CT-discograms showed an intraforaminal herniated disc (arrow) at the L5-S1 level. D: A CT-discogram obtained at L5-S1 showing partial resection in the L4 lamina (arrow). E: Postoperative 3-D reconstruction showing the oval fenestration in the L4 lamina.

**Figure 2:**
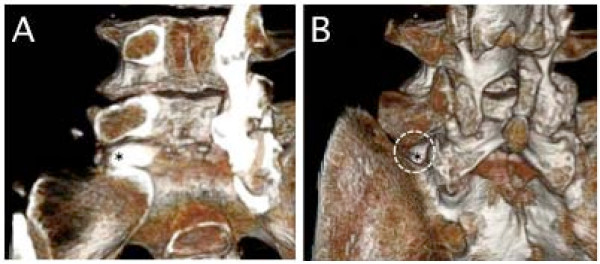
**Case 13. A three-dimensional CT-discogram obtained from a 78-year-old male who presented with severe left leg pain**. (A) The herniated disc (*) was located from the intraforaminal to the extraforaminal lesion. (B) A tubular retractor was set at the dotted area, and the herniated disc (*) was removed.

Posterior decompression was performed using a microendoscope in all patients. Fourteen patients underwent the extraforaminal approach [3] and 2 patients underwent the translaminar approach [6,7]. In addition to decompression of extraforaminal lesions, one patient also underwent intraspinal canal decompression because it was though likely that the intraspinal canal lesions could not be excluded prior to the index surgery. The METRx MED system (Medtronic Sofamor Danek) was used for the entire procedure. The patient was placed prone on a Hall frame. A skin incision of 2 cm was made about 2 to 5 cm lateral to the spinous process. After a muscle splitting approach using a series of sequential dilators, a tubular retractor with a diameter of 1.6 cm was set. For the translaminar approach, a small oval fenestration about 8 mm in the hemilamina, craniomedially to the facet joint, was performed (Figure 1E). For the extraforaminal approach, the muscles attached to the inferior transverse process and the lateral edge of the facet were removed, and the vertebral disc was isolated from the superior-medial portion of the inferior transverse process. In case of the extraforaminal herniation, the herniated disc may have protruded in this lesion (Figure 2B). The nerve root was also identified in this lesion or that just cranial to the disc. In case of a protruded disc, the disc just caudal to the nerve root was removed using curetts and a punch. No attempts were made to remove the osteophytes of the vertebral body. The patients were then encouraged to stand up and walk on the day after surgery.

### Preoperative and Postoperative Evaluations

The following items were investigated: 1) the preoperative radiological findings, including MRI and disco-CT scans; 2) intraoperative disc protrusion; and 3) the surgical outcomes evaluated using the JOA score for lower back pain (Table. 1).

**Table 1:** Criteria for the JOA scoring system

Subjective symptoms (9 points)	
low-back pain	
none	3
occasionally mild	2
always present of sometimes sever	1
always sever	0
leg pain &/or numbness	
none	3
occasionally mild	2
always present or sometimes sever	1
always sever	0
walking ability	
normal walking	3
able to walk > 500 m, pain/numbness/weakness present	2
unable to walk 500 m due to pain/numbness/weakness	1
unable to walk 100 m due to pain/numbness/weakness	0
objective finding (6 points)	
straight leg raising	
normal	2
30-70 degree	1
< 30 degree	0
sensory function	
normal	2
mild sensory disturbance	1
apparent sensory disturbance	0
motor function	
normal (MMT normal)	2
slight decrease muscle strength (MMT good)	1
marked weakness (Grade 3-0)	0
restriction of ADLs (14 points) †	
none	2
moderate	1
severe	0
bladder function (-6 points)	
normal	0
mild dysuria	-3
severe dysuria	-6
total score	29

* ADL = activities of daily living; MMT = manual muscle testing.

† ADL include the following; turning over while lying down, standing, washing one's face, leaning forward, ability to sit for approximately 1 hour, ability to lift or hold heavy objects, and ambulatory ability.

## Results

### Clinical Findings

The neurological findings during the physical exam varied in each patient (Table 2). The Kemp sign was positive in 13 (76%) patients. The nerve root stretching test (FNST or SLRT) was positive in 11 (65%) patients. In cases with L3 or L4 nerve root involvement, the femoral nerve stretching test was frequently positive.

**Table 2:** Patient characteristics and outcomes

	Age, Sex	Level	Kemp Sign	SLRT	FNST	Sensory Disturb	Muscle Weakness	disc protrusion CT discogram	Preop conservative treatment	disc protrusion op-finding	Preop JOA score	Postop JOA score	Recovery Rate (%)	different outcomes
1	88, F	L5-S1	+	-	-	L5	-	+	3 M	+	3	11	30.8	Contralateral side*
2	69, F	L3-L4	+	+	+	L4	IP, QF, EHL	+	5 M	+	7	14	31.8	Contralateral side
3	70, F	L5-S1	+	-	-	L5	-	-	2 M	-	12	12	0	no pain relief
4	80, F	L5-S1	+	-	-	L5	EHL	-	2 M	-	12	19	41.2	Ipsilateral side†
5	70, F	L5-S1	+	+	-	L5	EHL	+	5 M	+	11	20	50	
6	56, M	L4-L5	+	+	+	L4	-	+	14 M	+	15	29	100	
7	76, F	L3-L4	-	-	+	-	-	+	6 M	+	14	28	93.3	Ipsilateral side
8	75, F	L5-S1	+	-	-	L5	EHL	+	8 M	+	5	20	62.5	
9	67, M	L5-S1	+	+	-	L5	EHL	+	9 M	+	8	29	100	
10	60, M	L5-S1	+	+	-	L5	EHL	NA	4 M	+	11	27	88.9	
11	68, M	L5-S1	-	-	-	L5	-	+	5 M	+	11	21	55.6	
12	40, F	L5-S1	-	+	-	L5, S1	-	+	2 M	+	14	19	33.3	
13	78, M	L5-S1	+	-	-	L5	EHL	+	6 M	+	11	16	27.8	
14	66, M	L5-S1	-	+	-	L5	EHL	-	4 M	-	20	28	88.9	Ipsilateral side
15	63, F	L3-L4	+	+	-	L3	-	NA	7 M	+	13	20	43.8	
16	66, M	L3-L4	+	-	+	L3	IP, QF	+	4 M	+	13	20	43.8	
17	62, F	L4-L5	+	-	+	-	EHL	NA	18 M	+	4	11	28	
											10.8 ± 4.3	20.2 ± 6.2	54.1 ± 30.0	

* contralateral side = later recurrence of the same symptom on the contralateral side of the surgery

† ipsilateral side = later appearance of symptoms on the contralateral side of the surgery

### Radiological Findings

MR imaging was performed in all 17 cases. On MR images, central spinal canal stenosis was not evident in any patient. Parasagittal MRI at the foramen was useful in assessing the presence of foraminal stenosis by the obliteration of the normal increased signal intensity fat (Figure 1A). CT-discography was performed (Figure 1B, C) in 14 patients. Ten patients showed disc protrusion in the foraminal (2 cases) or extraforaminal (8 cases) lesion. A nerve root block with a local anesthesic immediately relieved the radicular pain in all 17 patients.

### Intraoperative disc protrusion

During the intraoperative observations, 13 patients were noted to have a protruding disc just caudal or anterior to the nerve root. Among these, 2 cases had a herniated disc just anterior to the nerve root, and the nerve root seemed wide and flat just anterior to the lateral facet, such that the herniated disc could only be found after exploration of the anterior lesion of the nerve root. In the cases with intraforaminal herniated disc removed by the translaminar approach, a herniated disc was found just caudal to the existing nerve root. Four cases had no disc protrusion, and only lateral fenestration, including the resection of some ligamentum flavum without discectomy, was performed.

The intraoperative findings with or without disc protrusion were compatible to the CT-discogram findings in all 14 cases who had undergone this examination.

### Surgical outcomes

Sixteen patients reported relief of their preoperative symptoms just after the surgery. One patient reported no pain relief. Three patients had later recurrence of the same symptoms on the ipsilateral side, and 2 patients had later appearance of symptoms on the contralateral side of the surgery. The mean JOA scores were 10.8 before surgery and 20.2 at the final follow-up, and the mean recovery rate was 54%.

No serious complications occurred in any of the patients during the surgical procedure. Two patients who had symptoms of remaining leg pain had revision surgery by PLIF.

## Discussion

The diagnosis of intraforaminal and extraforaminal nerve root compression is difficult to establish based on a single diagnostic modality. Comprehensive clinical and radiological information are required. Patients with this disease present with unilateral leg pain and demonstrate deficits of exiting nerve root function, including muscle weakness of the lower extremities. The Kemp sign, which induces a narrowing of the foraminal and extraforaminal area by forcing dorsolateral extension of the lower back, was positive in most patients.

Radiologically, parasagittal MRI at the foramen was useful for assessing the presence of foraminal stenosis by the obliteration of the normal increased signal intensity fat. In our series, all 17 patients had features of foraminal stenosis on MRI, however, it was difficult to determine whether the location of stenosis was intrafromainal or extraforaminal in many cases. The causes of stenosis in lateral lesions were a combination of protruded discs, degenerated ligamentum flavum, protruded osteophytes of the vertebral body, and degenerative facets. It was difficult to determine which element(s) had caused the nerve entrapment based on the findings of MRI alone.

**Figure 3:**
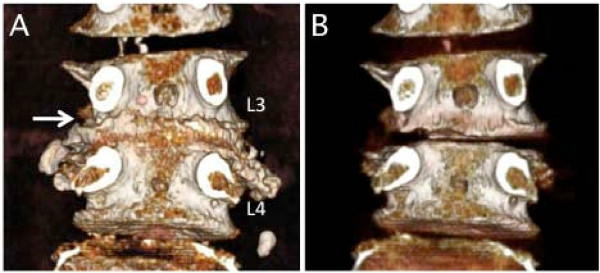
**Case 2. A three-dementional CT-discogram obtained from a 69-year-old female who presented severe left leg pain**. A protruded disc (A) was recognized by comparing the plane 3-D CT scan (B).

CT discography was therefore useful for elucidating the participation of the protruded disc for the nerve root entrapment at the lateral area. All cases that were speculated to have a protruded disc by CT discography also demonstrated corresponding surgical findings. The 3-D reconstruction of CT discograms could describe the protrusion of the vertebral disc, especially compared to the plane CT (Figure 3A, B). Three-dimensional reconstruction of CT discograms can also describe the positional relationship of the protruded disc to the facet, transverse process and pars (Figure 2A, B). These features were very useful for the planning of the surgical approach, especially since the endoscopic surgery can only expose a small posterior element of the lumbar spine.

There are several posterior decompression approaches for performing intraforaminal and extraforaminal nerve root compression at the lumbar spine. These include the interlamina approach [8], translamina approach [6,7] and extraforaminal approach [3,9-11]. For the interlamina approach, the performance of a medial facetectomy with laminotomy may provide an adequate exposure of medial foraminal lesions. However, this procedure is usually limited to the L5-S1 level because the lamina at other levels is not wide enough to perform a resection. For the translaminar approach, a small oval fenestration in the hemilamina, craniomedially to the facet joint, was performed. The translaminar approach is usually applied at either the L3-L4 or the L4-L5 level, because the lamina at L1 or L2 may be too thin to make an oval hole to preserve the pars interarticularis and the disc level at the L5-S1 level is usually too caudal for this approach. For the extraforaminal approach, the removal of the intertransversarious ligament may adequately expose the lateral compartment. At the L5-S1 level, since the space between the sacral ala and L5 transverse process is usually very narrow, the lateral edge of the L5-S1 facet joint and the superior-medial portion of the sacral ala were resected.

Surgical options for intraforaminal and extraforaminal nerve root compression at the lumbar spine include posterior decompression with or without fusion. The advantage of decompression without fusion is that it is less invasive. On the other hand, this type of procedure is demanding and limits the decompression so it cannot destroy the mechanical structures. In this study, we investigated the surgical results of posterior decompression alone using a microendoscope, and we found that posterior endoscopic decompression alone could improve radicular pain in most patients for at least a short period of time after surgery.

Problems associated with posterior decompression without fusion still remain, including the recurrence of symptoms on ipsilateral or contralateral sides at the same vertebral level. These late-occurred symptoms are speculated to be caused by the foraminal stenosis. It is not easy to differentiate intraforaminal and extraforaminal stenosis, or to determine whether there is a combination of intraforaminal and extraforaminal compression before surgery. Even though CT discography is useful for detecting disc protrusion, the other foraminal stenosis factors, such as the degeneration of the ligamentum flavum, could not be detected. Endoscopic decompression surgery is an appropriate less-invasive surgical option for lateral root entrapment in older patients, however, there may be limitations to using only decompression surgery, and posterior interbody fusion with decompression may therefore be the treatment of choice when the foraminal stenosis is suspected before surgery.

## Competing interests

The authors declare that they have no competing interests.

## Authors' contributions

TD has contributed to the conception and design of the study, performing surgeries, acquisition of data, analysis and interpretation of data, and drafted the manuscript. KH, YM, OT and KT performed part of literature review and acquisition of data. YI participated in the design and coordination and helped to draft the manuscript. All authors read and approved the final manuscript.
